# DLST drives lung adenocarcinoma progression by modulating cuproptosis-related copper homeostasis and apoptosis via an EP300-dependent mechanism

**DOI:** 10.1016/j.tranon.2026.102975

**Published:** 2026-08-14

**Authors:** Tonghai Huang, Lin Chen, Zeyao Li, Xiean Ling, Kangqi Ren

**Affiliations:** Department of Thoracic Surgery, Shenzhen People’s Hospital, 1st Affiliated Hospital of Southern University of Science and Technology, 2nd Clinical Medical College of Jinan University, Shenzhen, 518020, Guangdong, China

**Keywords:** Lung adenocarcinoma, Cuproptosis, Dihydrolipoamide S-succinyltransferase (DLST), Dihydrolipoamide dehydrogenase (DLD), E1A binding protein p300 (EP300)

## Abstract

**Background:**

Dihydrolipoamide S-succinyltransferase (DLST), a key enzyme in the tricarboxylic acid cycle, has recently been implicated in cuproptosis. However, its role in lung adenocarcinoma (LUAD) remains unclear.

**Methods:**

DLST expression and clinical relevance were analyzed using TCGA and GEO datasets. Functional assays, including western blotting, flow cytometry, and copper ion detection, were performed in LUAD cell lines. *In vivo* tumor models were established to evaluate tumor growth. Bioinformatics analyses, co-immunoprecipitation, and single-cell RNA sequencing were conducted to investigate upstream and downstream regulatory mechanisms.

**Results:**

DLST was significantly upregulated in LUAD tissues and was associated with advanced stage and poor prognosis. Mechanistically, upstream, EP300 interacted with DLST and enhanced its phosphorylation. At the core, DLST overexpression increased intracellular copper levels, upregulated the cuproptosis-related proteins HSP70 and SLC31A1, and inhibited apoptosis, these effects were partially reversed by copper chelation. Downstream, DLST exerted anti-apoptotic effects through upregulation of DLD. *In vivo* experiments confirmed that DLST promoted tumor growth. Single-cell analysis further revealed co-expression of DLST and EP300 in immune-related cell populations, suggesting a potential role of this axis in the tumor microenvironment.

**Conclusion:**

DLST promotes LUAD progression by regulating cuproptosis and apoptosis through the EP300-DLST-DLD axis, suggesting that this axis may represent a candidate for future therapeutic exploration in LUAD.

## Highlights

• DLST is significantly upregulated in lung adenocarcinoma (LUAD) and correlates with advanced clinical stage and poor prognosis, promoting tumor growth by increasing intracellular copper levels and modulating cuproptosis-related proteins (HSP70, SLC31A1).
• Mechanistically, the EP300–DLST–DLD axis is identified as a key pathway where EP300 enhances DLST phosphorylation, and DLST exerts anti-apoptotic effects via upregulation of DLD, linking cuproptosis regulation to tumor progression.
• Targeting DLST or the EP300–DLST–DLD axis represents a potential therapeutic strategy for LUAD, supported by *in vivo* tumor models and single-cell RNA-seq revealing co-expression in the tumor microenvironment.

## Graphical abstract

**Figure:**
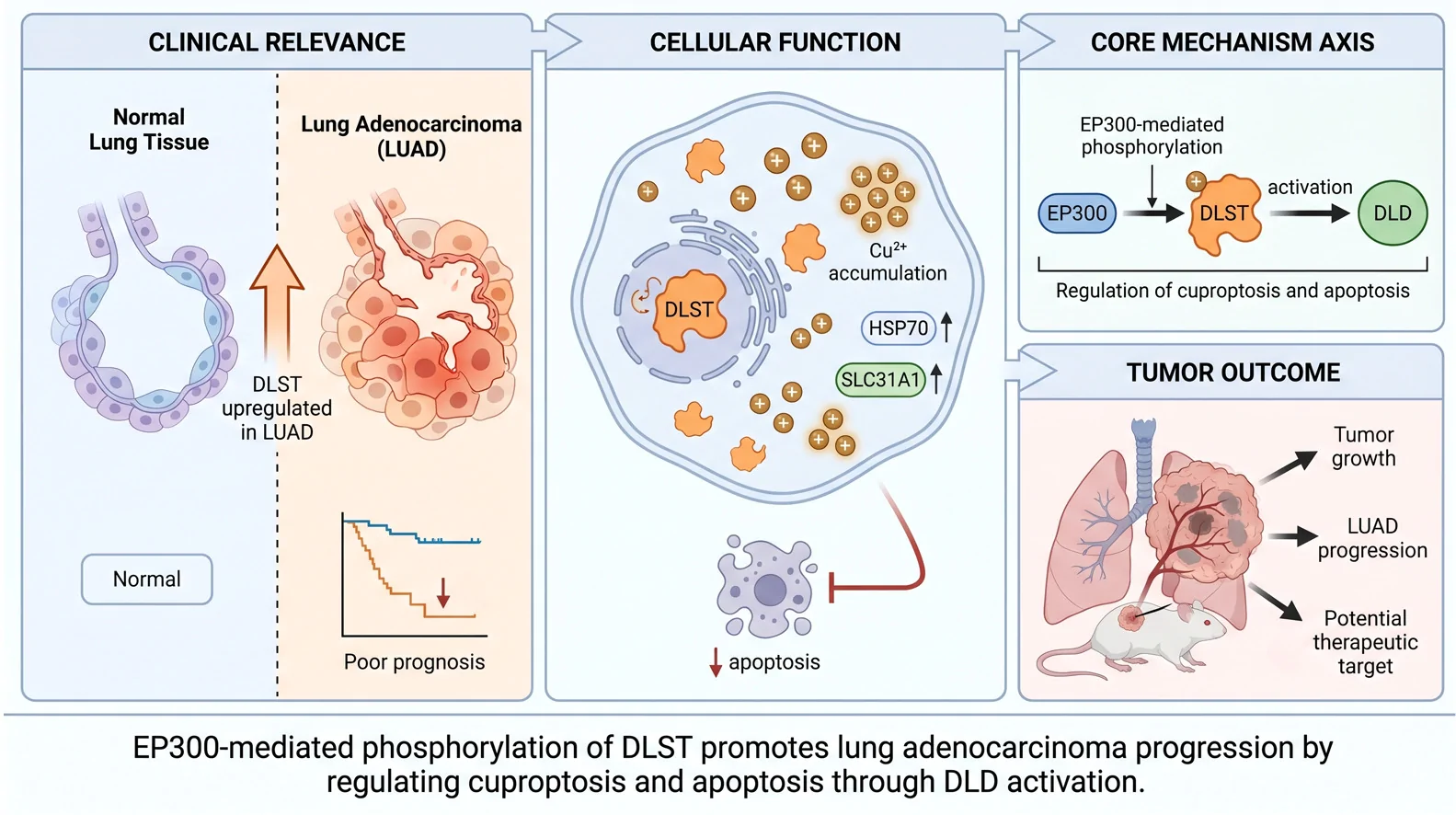

## Introduction

Among all malignancies, lung cancer remains one of the most commonly diagnosed malignancies and is associated with poor survival outcomesemerging research hotspot such as m6A modifications, non-coding RNAs, tumor microenvironment, and immune evasion mechanisms, posing a significant threat to human health [1]. As a major histological variant of NSCLC, lung adenocarcinoma (LUAD) demonstrates particularly high prevalence [2]. LUAD exhibits complex and diverse subtypes, such as squamous, micropapillary, and acinar subtypes, which are closely associated with patient prognosis. This diversity poses significant challenges for the treatment of LUAD [[3], [4], [5], [6]]. Therefore, exploring the molecular mechanisms underlying LUAD development can enhance our understanding of its progression and enable targeted interventions at critical steps, ultimately advancing precision medicine. Current research has identified that the pathogenesis of LUAD involves multiple driver gene mutations (e.g., EGFR, KRAS, ALK), as well as emerging research hotspot such as m6A modifications, non-coding RNAs, tumor microenvironment, and immune evasion mechanisms [[7], [8], [9],^10^].

In recent years, the role of cuproptosis in diseases has garnered increasing attention. Cuproptosis is a mitochondria-dependent form of programmed cell death that involves the interference of Cu²⁺ with lipidated proteins, leading to toxicity [11]. Key molecular players or enzymes in the cuproptosis pathway include ferredoxin 1(FDX1), lipoyl synthase (LIAS), dihydrolipoamide S-acetyltransferase (DLAT), and solute carrier family 31 member 1 (SLC31A1). SLC31A1 serves as the "switch" initiating cuproptosis, encoding a Cu²⁺ transporter that regulates the primary pathway for copper entry into cells [12]. FDX1, upstream in the pathway, reduces Cu²⁺ to Cu⁺, enhancing Cu⁺ activity and driving the lipidation of enzymes such as DLAT in the tricarboxylic acid (TCA) cycle. This process induces stress responses and cell death [13,14]. Additionally, methylation modifications of FDX1 have been found to promote cuproptosis in gastric cancer cells, thereby influencing cancer progression [15]. LIAS, a mitochondrial fatty acid synthase, catalyzes the synthesis of lipoic acid (LA) and facilitates the binding and aggregation of Cu⁺ with lipoic acid. These aggregates disrupt mitochondrial metabolism, trigger stress responses, oxidative reactions, and inactivation of iron-sulfur cluster proteins, ultimately leading to cell death [16,17]. DLAT acts as a direct target of Cu⁺, binding to monovalent Cu⁺ and thereby interfering with mitochondrial metabolism, resulting in apoptosis [18].

Dihydrolipoamide S-succinyltransferase (DLST) is a core metabolic enzyme in the TCA cycle and plays a significant regulatory role in the energy metabolism reprogramming of cancer cells. Studies have shown that in triple-negative breast cancer, DLST expression is significantly associated with poor patient prognosis and survival outcomes, and its deletion promotes apoptosis in breast cancer cells [19]. In neuroblastoma, DLST expression is also closely linked to poor prognosis and disease severity. Furthermore, research has revealed that DLST promotes neuroblastoma progression via its role in NADH oxidative phosphorylation [20]. In colorectal cancer, DLST expression is significantly reduced in tumor tissues, and patients with high DLST expression exhibit better prognoses [21]. Additionally, DLST has been implicated in copper-induced death. When lipidated DLST interacts with Cu⁺ under high copper conditions, it induces protein aggregation and mitochondrial stress, leading to programmed cell death [14]. In a risk prediction model for non-small cell lung cancer, DLST was identified as a key gene in cuproptosis and is associated with patient prognosis and metastasis [22]. However, the biological function of DLST in LUAD remains elusive, and its potential role in regulating LUAD cell processes is yet to be determined.

Overall, this study aims to employ a combination of bioinformatics approaches *in vitro* and *in vivo* experiments to investigate and validate the biological functions of DLST as well as its role in the process of cuproptosis in LUAD and the hypothesis that DLST overexpression promotes LUAD progression through a coordinated EP300–DLST–DLD axis. By conducting differential expression analysis, weighted gene co-expression network analysis (WGCNA), and gene-gene interaction (GGI) analysis, we will screen regulatory molecules upstream and downstream of DLST. Additionally, we will validate its regulatory mechanisms through techniques such as western blot and flow cytometry. This study aims to provide a theoretical basis for the early diagnosis.

## Materials and methods

### Materials

Cell Copper (Cu) Content Detection Kit (JL-T2630) was purchased from JONLNBIO (Shanghai, China). Lipo8000(C0533-7.5 ml) and Immunoprecipitation kit (Protein A+G magnetic bead method) (P2179) were purchased from Beyotime Biotechnology (Shanghai, China). Annexin V-FITC/PIApoptosis Detection Kit was purchased from YEASEN (40302ES20, Shanghai, China). Overexpression lentivirus was synthesized by Sangon Biotech (Shanghai, China). Opti-MEM was purchased from yuanye Bio-Technology Co., Ltd (Shanghai, China). Rabbit anti-Homo sapiens (Human) HSP70 Polyclonal antibody (#4872T), SLC31A1 Polyclonal antibody (#13086S), DLST Polyclonal antibody (#5556S), EP300 Polyclonal antibody (#86377T) were bought from CST (Massachusetts, USA). DLD rabbit polyclonal antibody (CSB-PA006928LA01HU) was bought from CUSABIO (Wuhan, China). Mouse Anti-β-Actin antibody (MFCD00164531) was obtained from Merck KGaA (Darmstadt, Germany). Nude mice were obtained from Shulaibao (Wuhan, China). Mogengel Matrix was (AC-M082724-10 ml) purchased from ACROBiosystems (Beijing, China). A549 cell line (KGG3215-1) and H1299 cell line (KGG3216-1) were obtained from Key GENE Bio TECH (Jiangsu, China). Tetrathiomolybdate (TTM) (HY-128530) was purchased from MCE (New Jersey, USA). Anti-Mouse&Rabbit Light chain for IP (001-180-005) and Anti-Mouse&Rabbit Heavy chain for IP (001-190-005) were bought from AlpVHHS (Chengdu, China).

### Cell culture and lentiviral transduction

A549 cells and H1299 cells were maintained in F-12 K or RPMI 1640 medium containing 10% heat-inactivated fetal bovine serum. Incubation occurred at 37 °C in a humidified atmosphere supplemented with 5% CO₂. To investigate the functional roles of DLST, DLD, and EP300 in lung adenocarcinoma cells, we generated cell lines with stable overexpression of each gene using lentiviral transduction (DLST-OE, DLD-OE and EP300-OE). Cells in the logarithmic growth phase were seeded into 24-well plates at approximately 50% confluency in 100 μL of complete medium per well and incubated overnight to allow adherence. Parallel wells of HEK293T cells were also seeded as controls. Optimal infection was performed when target cells reached ∼70% confluency. On the second day, lentiviral particles stored at 4 °C were gently mixed before use. If frozen at −80 °C, the virus was thawed on ice and mixed thoroughly. Before infection, the cell morphology and adherence were checked to ensure good condition. Following aspiration of the culture medium, the cells underwent two washes with phosphate-buffered saline (PBS). Fresh complete medium was added if needed, particularly when contamination or floating cells were observed. A defined volume of virus was added directly to the A549 and H1299 cells. The virus-containing medium was gently mixed and the plate was incubated at 37 °C with 5% CO₂. After 24 h, the medium containing virus was discarded, and cells were replenished with fresh complete medium. The incubation continued for an additional 24–48 h. Between 48 and 72 h after infection, selection was initiated by adding puromycin at a concentration of 2 μg/mL, with the optimal concentration determined by prior titration experiments or literature reference. Cells were cultured in puromycin-containing medium for 3–7 days until non-infected control cells were fully eliminated. Surviving cells were expanded, passaged, and maintained in selection medium for at least three passages to establish stable overexpression. Successfully transduced cell lines were cryopreserved for future use.

### Western blot analysis

Cell lysis utilized RIPA buffer containing protease/phosphatase inhibitors. Following 30 min on ice, lysates underwent centrifugation. Supernatant collection preceded protein quantification via BCA assay. Equal protein aliquots (30 µg) were combined with 5 × SDS buffer, denatured (100 °C, 5 min), and electrophoresed on 8–12% SDS-polyacrylamide gels. Proteins were electroblotted onto PVDF membranes. After blocking (5% non-fat milk/TBST, 1 h RT), membranes received primary antibodies (4 °C overnight), then HRP-linked secondary antibodies (1 h RT). ECL detection visualized bands, β-actin served as control. The experiment was conducted three times. The relative protein expression level was calculated as the ratio of the target protein band intensity to the corresponding β-actin band intensity, and the values were normalized to the vector control group. All western blot experiments were performed with 3 independent biological replicates.

### Flow cytometric analysis of apoptosis

A549 and H1299 cells stably transduced with empty vector (vector) or DLST-overexpression lentivirus (DLST-OE) were seeded at 6-well plates and cultured overnight. DLST-OE cells were treated with the copper chelator Tetrathiomolybdate (TTM) at 20 mM for 12 h, an equivalent volume of DMSO was added to the vector and untreated DLST-OE groups as a control. At the end of treatment, cells were harvested with trypsin-EDTA, washed twice with ice-cold PBS, and stained with Annexin V-FITC and propidium iodide (PI) according to the manufacturer's instructions. After a 15 min incubation at room temperature in the dark, samples were analyzed on a flow cytometer. For each sample, at least 10,000 single cells were acquired. All experiments were performed with 3 independent biological replicates, each conducted on a separate day. Group comparisons among vector, DLST-OE, and DLST-OE+TTM were analyzed by two-way ANOVA.

### Copper (Cu) content detection

Intracellular copper levels were measured using the Cell Copper (Cu) Content Detection Kit (JL-T2630, JONLNBIO, Shanghai, China) according to the manufacturer's instructions. A549 and H1299 cells were seeded at 6-well plates and cultured overnight. The following day, cells were treated with TTM at 20 mM or an equivalent volume of DMSO for 12 h. Cells were then harvested, transferred to centrifuge tubes, washed twice with ice-cold PBS, and pelleted by centrifugation. Cell pellets were resuspended in distilled water at a density of 5 × 10^6^ cells per 0.4 mL. Cell disruption was performed by ultrasonic homogenization on ice. Lysates were centrifuged at 12,000 × *g* for 10 min at 4 °C, and the supernatant was collected for analysis. Copper content was determined in a 96-well plate format by measuring absorbance at 600 nm and calculated from a copper standard curve provided in the kit. Results were normalized to cell number per 10^6^ cells/ total protein concentration determined by BCA assay. Each condition was tested in 3 independent biological replicates. Group comparisons among vector, DLST-OE, and DLST-OE+TTM were analyzed by two-way ANOVA.

### Orthotopic implantation model

Immunocompromised nude mice aged 4–6 weeks (*n* = 6) were maintained under SPF conditions with ad libitum access to rodent chow and drinking water. A549 cells were collected during the logarithmic growth phase, and at a density of 10 million cells per milliliter, the cells were suspended in medium lacking serum. Isoflurane anesthesia was administered to the mice, and the left chest was disinfected with 75% ethanol. A small skin incision (approximately 5 mm) was made between the fourth and fifth ribs to expose the lung. Using a 29-gauge needle, 50 μL of the cell suspension (containing 5 × 10⁵ cells) was slowly injected into the left lung parenchyma. The injection site was gently compressed with sterile gauze to prevent leakage. The incision was closed with surgical glue or absorbable sutures. Mice were monitored daily for signs of distress or dyspnea. Mice showing weight loss exceeding 20% of initial body weight, or inability to access food and water were humanely euthanized. After 3–4 weeks mice were euthanized by cervical dislocation under deep isoflurane anesthesia. Tumor weight was measured using an electronic balance, and tumor volume was calculated using the formula: V = (length × width^2^) / 2. All procedures were approved by the Ethics Committee of Shenzhen People’s Hospital.

### Co-Immunoprecipitation (Co-IP)

Cells from each treatment group were collected, and total proteins were extracted according to the immunoprecipitation kit instructions. For input samples, protein lysates were mixed with SDS loading buffer in a new EP tube, thoroughly vortexed, boiled at 95–100 °C for 10 min, cooled to room temperature, and centrifuged for 5 min. IP samples were prepared following the manufacturer’s protocol. The samples were then subjected to Western blotting analysis.

### Chromatin immunoprecipitation (CHIP)

Cells (vector control and EP300-OE) were cross-linked with 1% formaldehyde for 10 min at room temperature and quenched with 125 mM glycine for 5 min. Cells were lysed in SDS lysis buffer and chromatin was sheared to 200–500 bp fragments by sonication on ice. Sheared chromatin was diluted in ChIP dilution buffer and incubated overnight at 4 °C with 5 μg of anti-EP300 antibody or normal rabbit IgG as a negative control. Immune complexes were captured with Protein A/G magnetic beads for 4 h at 4 °C, followed by sequential washes with low-salt, high-salt, LiCl, and TE buffers. Cross-links were reversed by incubation at 65 °C overnight, and DNA was purified using a DNA purification kit. Purified DNA was analyzed by quantitative PCR using primers targeting the predicted EP300-binding region within the DLST promoter. Enrichment was calculated as the percentage of input DNA or as fold enrichment relative to the IgG control. Each experiment was performed with at least three biological replicates.

### Data collection

We obtained the transcriptome data and clinical data of lung adenocarcinoma (TCGA-LUAD) from the Cancer Genome Atlas (TCGA) data portal https://Xena.UCSC.edu/ through UCSE XEAN https://portal.gdc.Cancer.gov. The GSE40791 gene expression dataset was obtained from the GEO database https://www.ncbi.nlm.nih.gov/geo/.

TCGA-LUAD transcriptome data: It contains a total of 600 samples, among which 541 are tumor samples (90.2%) and 59 are normal samples (9.8%), used for DESeq2 differential expression analysis. After taking the intersection of this dataset and TCGA-LUAD survival data (a total of 646), 576 samples were obtained, including 517 tumor samples and 59 normal samples. This intersection data is used for subsequent KM survival curve and Cox regression analysis.

TCGA-LUAD clinical data: The clinical data was downloaded from the TCGA GDC official website. The original file contains 721 patient records. Clinical stage: Stage I (188), Stage II (85), Stage III (61), and Stage IV (22). T stage: T1 (104), T2 (204), T3 (30), and T4 (18). N stage: N0 (227), N1 (75), N2 (53), and N3 (1). M stage: M0 (334) and M1 (22). Age: younger than 65 years old (160), 65 years old or older (196). Gender: female (179), male (177).

GEO differential expression validation dataset: GSE19188 contains 156 samples, including 65 normal samples and 91 tumor samples. GSE40791 contains a total of 194 samples, including 100 normal samples and 94 tumor samples.

GEO prognosis validation dataset: GSE13213 contains 117 samples, and GSE31210 contains 226 samples.

### Differential expression of DLST gene and its prognostic analysis

Perform differential expression analysis using DESeq2. Using the median expression value of DLST in TCGA-LUAD tumor samples as the boundary, the samples were divided into the DLST high-expression group (High) and the low-expression group (Low), and the differentially expressed genes between the two groups were compared. The filtering criteria for differentially expressed genes were |log₂ fold change| > 0.2 and *P*〈 0.05. Additionally, the differential expression thresholds of DLST between tumor and normal tissues in the external validation datasets GSE19188 and GSE40791 were set as |log₂FC| 〉 0.5 and *P* value < 0.05.

Based on the expression profile data of TCGA-LUAD tumor samples, a co-expression network was constructed using the WGCNA package. The soft threshold was selected by the pickSoftThreshold function, and the optimal soft threshold (power = 7) was determined based on the scale-free topology model fit index (R²) ≥ 0.85. The network construction parameters are as follows: the TOM type is unsigned, the minimum number of module genes (minModuleSize) is 100, and the module merging and cutting height (mergeCutHeight) is 0.25. The module characteristic genes (module eigengene) were correlated with the high/low expression of DLST trait, and the module-trait correlation coefficient and the corresponding significance P value were calculated. The screening criterion for significant associated modules was *P* < 0.05. The genes in the modules significantly associated with the DLST group were selected as the key genes of the module, and the intersection with the differentially expressed genes was taken to obtain the final key gene set.

The Wilcoxon rank test was applied for two analyses: (1) comparing DLST expression in tumor versus normal groups, and (2) examining potential links between expression patterns and pathological characteristics such as gender, age, clinical stage and survival prognosis.

### Single-cell data analysis

For processing of the GSE131907 scRNA-seq dataset, we utilized version 4.3.0 of the Seurat R package. Low-quality cells with > 5% of the number of mitochondrial genes were excluded. Cell markers and cell type annotations were used according to the methods established by TISCH.

### Combined model score for EP300-DLST-DLD

For TCGA-LUAD, the script reads the TPM expression matrix and overall survival clinical information, standardizes the sample barcode format, retains the primary tumor samples, and if a patient has multiple primary tumor samples, takes the average expression value of the three genes as the expression value at the patient level. The expression matrix automatically determines whether a log2(x + 0.1) transformation is needed based on the distribution to reduce the impact of different expression magnitude on the score construction. Within each cohort, the expression values of EP300, DLST, and DLD are z-score standardized separately to obtain EP300_z, DLST_z, and DLD_z. The non-weighted combined score is defined as the sum of the three: axis_score = EP300_z + DLST_z + DLD_z. Except for the weighted score, the script also fits the Cox proportional hazards model of Surv(Time, Event) ∼ EP300_z + DLST_z + DLD_z in the TCGA-LUAD cohort, extracts the Cox regression coefficients of the three genes as weights, and constructs the Cox weighted combined score: axis_score_weighted = βEP300 × EP300_z + βDLST × DLST_z + βDLD × DLD_z. The high and low score groups are determined using the optimal cut-off method, using survminer:surv_cutpoint, and setting minprop = 0.30 to ensure that each group has no <30% of the samples. After grouping, Kaplan-Meier curves, log-rank tests, and Cox models are used to calculate the HR and 95% CI of the high-score group relative to the low-score group in TCGA-LUAD. In TCGA-LUAD, further single-factor Cox, multi-factor complete Cox, and stage grouping Cox are conducted. The complete model includes axis score, age, gender, Stage, T stage, N stage, and M stage, and the stage grouping model defines Stage I-II as Early and Stage III-IV as Advanced, and simultaneously includes axis score, age, gender, and Stage_group.

### Signal pathway scoring

To evaluate the functional orientation of the combined model score, in the GSE13213 cohort, pathway activity scores were calculated for five gene sets related to the DLST-DLD metabolic axis, namely copper death (Cuproptosis), TCA cycle, Pyruvate metabolism, Protein lipoylation, and Oxidative phosphorylation. The pathway scores were compared between the high and low axis score groups using the Wilcoxon rank sum test. Additionally, Spearman correlation analysis was used to assess the correlation between consecutive axis scores and each pathway score, and a correlation coefficient bar chart was drawn.

### Data analysis

The statistical environment R (v4.2.1) was employed for data analysis. Intergroup differences were assessed by one-way ANOVA or Student's *t*-test. For survival data analysis, we applied Kaplan-Meier methodology with log-rank tests or Cox regression models. A significance threshold of *P* < 0.05 was established (*P < 0.05, **P < 0.01, ***P < 0.001; ns = not significant)

## Results

### DLST is highly expressed in lung adenocarcinoma tumor tissue and associated with prognosis in patients

**Fig. 1:**
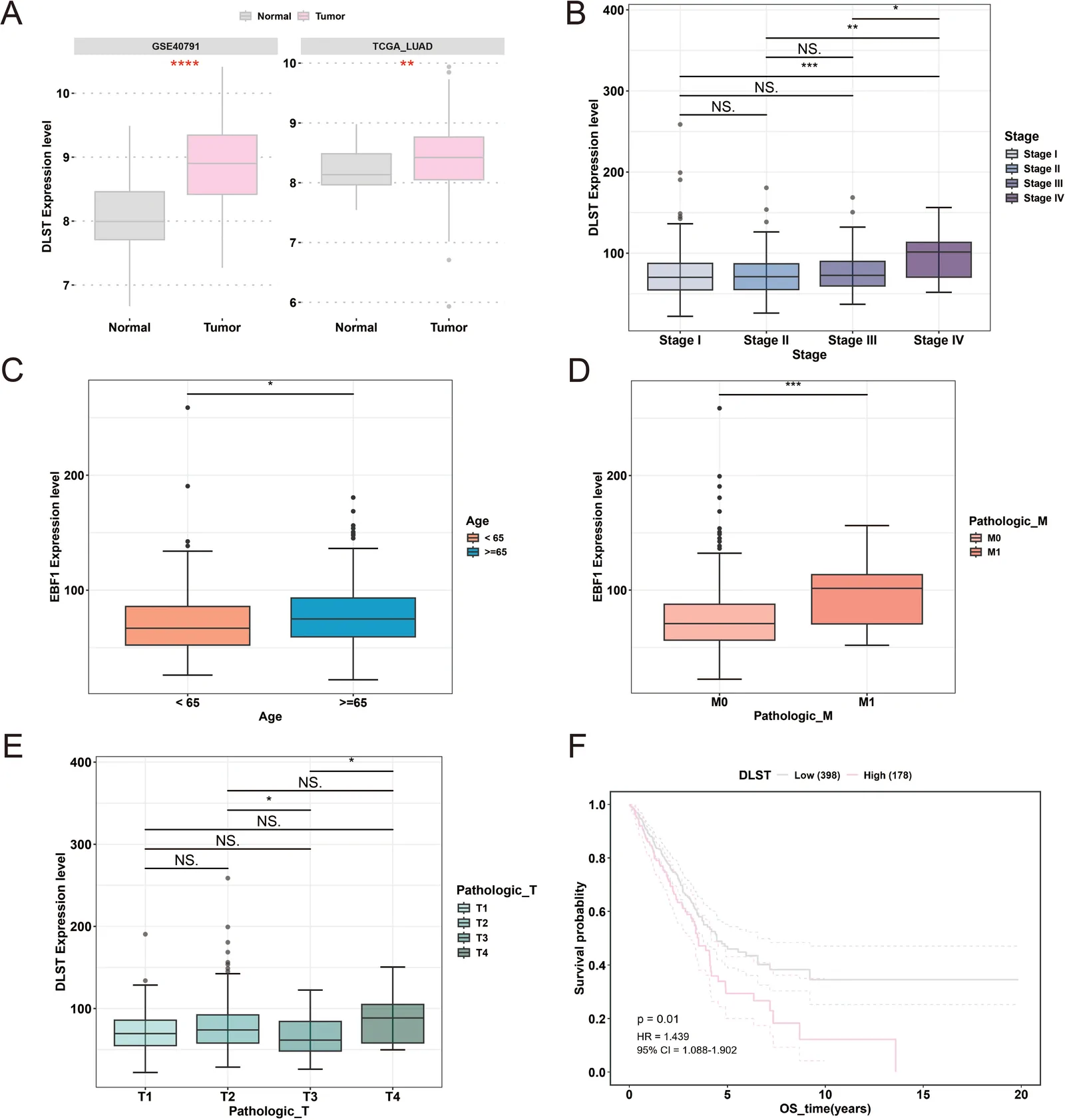
DLST is highly expressed in lung adenocarcinoma tumor tissue and associated with poor prognosis in patients. (A) DLST mRNA expression in LUAD tumor versus adjacent normal tissues in TCGA-LUAD (*n* = 541 tumor vs. *n* = 59 normal) and GSE40791 (*n* = 94 tumor vs. *n* = 100 normal). (B–E) Associations between DLST expression and clinicopathological features in TCGA-LUAD. (B) pathologic stage (Stage I *n* = 188, Stage II *n* = 85, Stage III *n* = 61, Stage IV *n* = 22), (C) age (<65 years *n* = 160, ≥65 years *n* = 196), (D) M stage (M0 *n* = 334, M1 *n* = 22), and (E) T stage (T1 *n* = 104, T2 *n* = 204, T3 *n* = 30, T4 *n* = 18). (F) Kaplan–Meier overall survival analysis of TCGA-LUAD patients stratified by DLST median expression (high *n* = 259, low *n* = 258). *P < 0.05, **P < 0.01, ***P < 0.001, ****P < 0.0001.

Initial investigation of DLST's involvement in LUAD involved comparative expression analysis of cancerous and non-cancerous samples from TCGA and GEO repositories. Both datasets (TCGA and GSE40791) confirmed markedly increased DLST expression in tumor tissues compared to their normal counterparts (*P*<0.01). (Fig. 1A). Further analysis of DLST expression levels across different clinical stages revealed that stage IV patients exhibited significantly higher DLST expression compared to stages III, II, and I (*P*<0.05) (Fig. 1B). Age-related analysis indicated that DLST expression levels were significantly higher in patients aged ≥65 years than in those younger than 65 years (*P*<0.05) (Fig. 1C). Additionally, M1-stage patients displayed significantly higher DLST expression compared to M0-stage patients (*P*<0.001) (Fig. 1D). Notably, DLST expression levels were significantly higher in T2 and T4-stage patients compared to T3-stage patients (*P*<0.05) (Fig. 1E). Kaplan-Meier survival analysis demonstrated that higher DLST expression was associated with lower patients’ survival rates (*P* = 0.01) (Fig. 1F). These findings indicate that DLST exhibits differential expression between lung adenocarcinoma and normal tissues and is correlated with patients’ poor prognosis, suggesting its potential involvement in the progression of LUAD.

### DLST overexpression inhibits apoptosis in lung adenocarcinoma cells

**Fig. 2:**
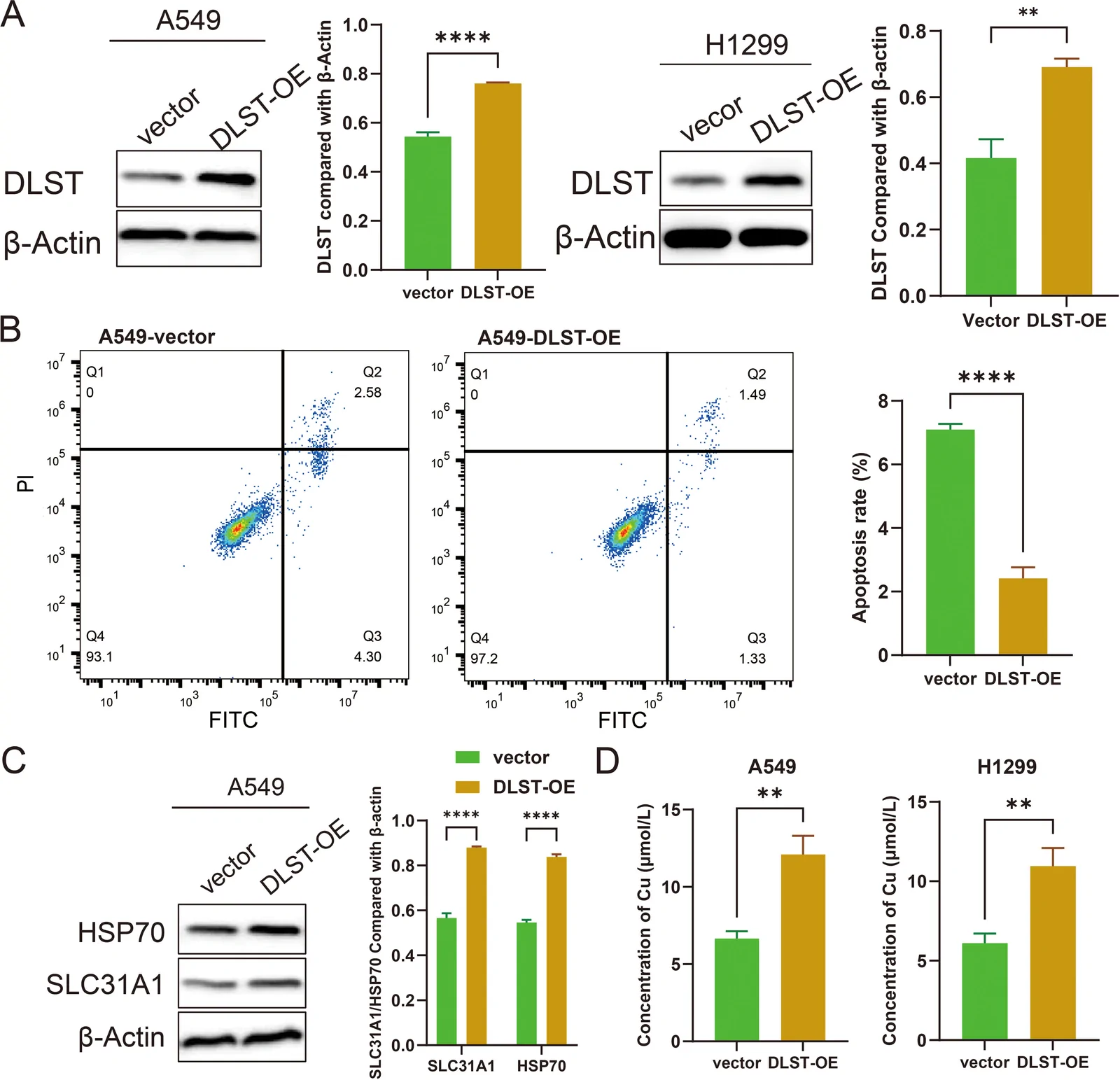
DLST overexpression inhibits apoptosis in lung adenocarcinoma cells. (A) Western blot confirmation of DLST overexpression (DLST-OE) versus empty vector control. (B) Flow cytometric quantification of total apoptotic cells (Annexin V-positive/PI-positive plus Annexin V-positive/PI-negative) in vector and DLST-OE cells. (C) Western blot analysis of HSP70 and SLC31A1 protein expression. (D) Intracellular copper content measured by the JONLNBIO copper detection kit. Data are mean ± SD from *n* = 3 independent biological replicates. Comparisons between vector and DLST-OE were performed using Student’s *t*-test. *P < 0.05, **P < 0.01, ***P < 0.001, ****P < 0.0001 vs. vector group.

Previous studies have demonstrated that DLST is involved in cuproptosis. In this study, we established a DLST-overexpressing (DLST-OE) LUAD cell line and confirmed its successful construction *via* western blot (*P*<0.01) (Fig. 2A). We first assessed the effect of DLST overexpression on cell apoptosis using flow cytometry, which revealed that DLST overexpression significantly suppressed apoptosis in LUAD cells (*P*<0.0001) (Fig. 2B). Subsequently, we compared the expression levels of cuproptosis-related proteins heat shock protein70 (HSP70) and SLC31A1 between the DLST-OE group and the vector control group. The results showed that DLST overexpression led to significantly higher protein expression levels compared to the control (*P*<0.0001) (Fig. 2C). To further confirm cuproptosis induction, we analyzed Cu levels in both groups. The DLST-OE group exhibited higher Cu levels compared to the vector group (*P*<0.01) (Fig. 2D).

### DLST overexpression alters cuproptosis-related protein expression and intracellular copper homeostasis in lung adenocarcinoma cells

**Fig. 3:**
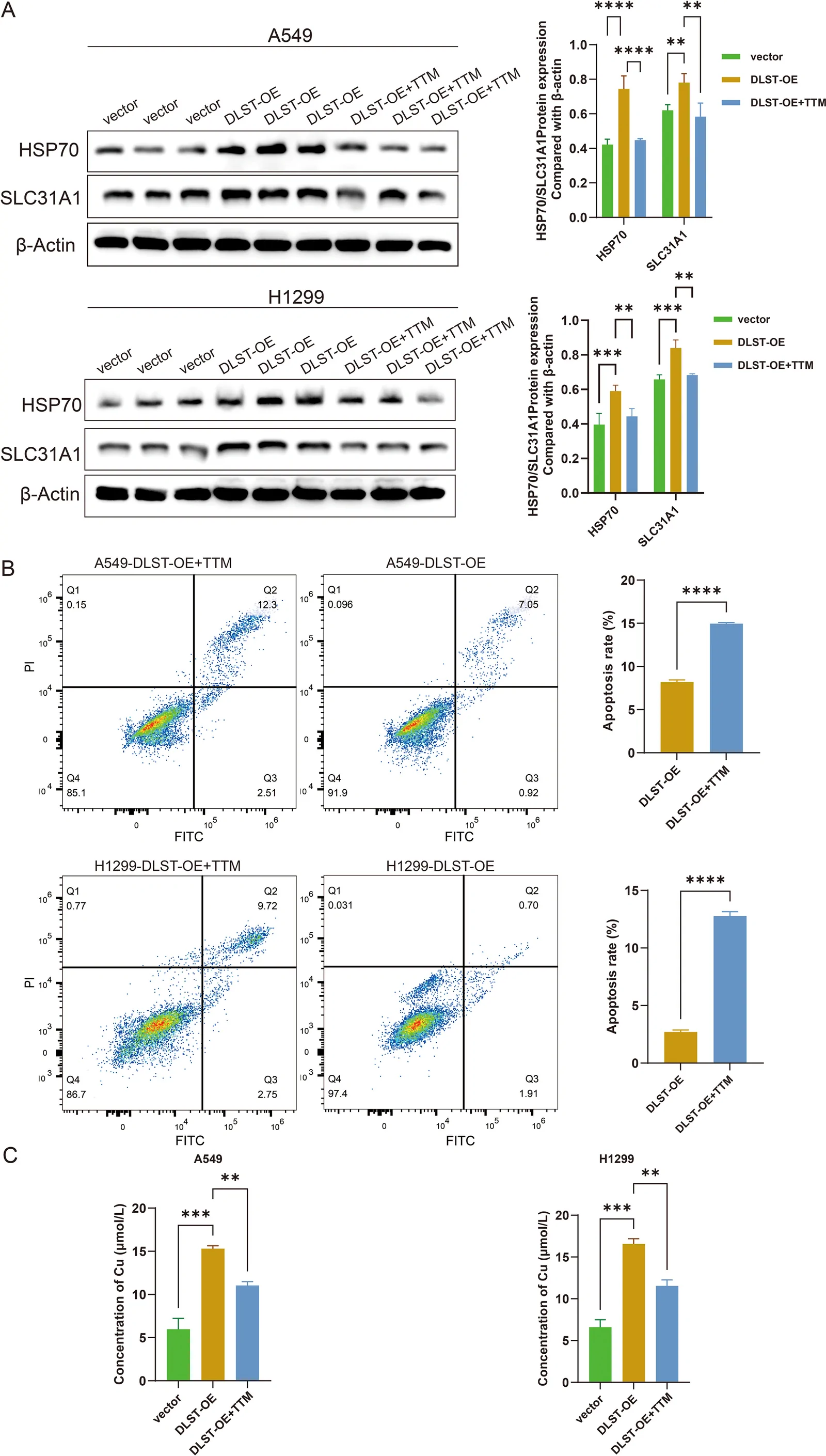
DLST overexpression alters cuproptosis-related protein expression and intracellular copper homeostasis in lung adenocarcinoma cells. (A) Western blotting was used to analyze the expression levels of HSP70 and SLC31A1 proteins in A549 and H1299 cells. (B) Flow cytometry was used to analyze apoptosis in A549 and H1299 cells under specified conditions. (C) Intracellular copper concentrations in A549 and H1299 cells were measured under specified treatment conditions. *P < 0.05, **P < 0.01, ***P < 0.001, ****P < 0.0001 vs. vector group or DLST-OE group.

In A549 and H1299 cells, DLST overexpression significantly upregulated the expression levels of HSP70 and SLC31A1 proteins, while treatment with the copper chelator TTM (DLST-OE+TTM) partially reversed this effect (*P*<0.01) (Fig. 3A). Flow cytometry results showed that the proportion of apoptotic cells in the DLST-OE+TTM group was significantly increased compared with the DLST-OE group (*P*<0.0001) (Fig. 3B). Further analysis revealed that DLST overexpression significantly increased the intracellular copper ion concentration, while TTM treatment significantly reduced the copper ion level (*P*<0.01) (Fig. 3C). These results indicate that DLST overexpression upregulates cuproptosis-associated proteins (HSP70 and SLC31A1) and increases intracellular copper levels, yet functionally suppresses apoptosis in LUAD cells. Copper chelation with TTM reversed both the marker upregulation and the anti-apoptotic effect, suggesting that DLST-mediated pro-survival signaling is copper-dependent. Collectively, these data support that DLST increases intracellular copper and the expression of copper-handling proteins, but the functional outcome is not cell death. Instead, DLST couples these copper-related changes to an anti-apoptotic program, at least in part through DLD, thereby promoting LUAD cell survival.

### Overexpression of DLST increased tumor growth in mice

**Fig. 4:**
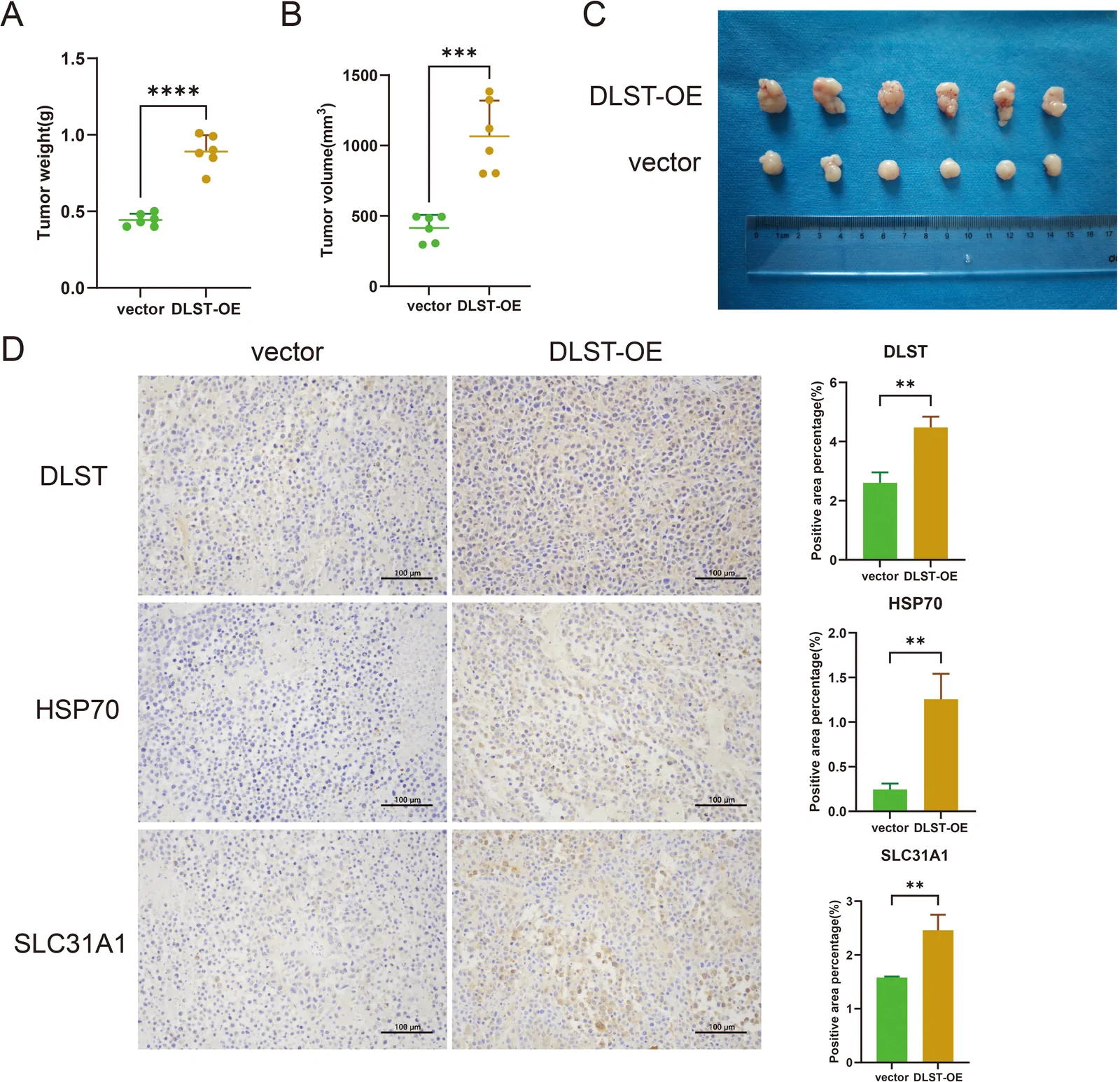
Overexpression of DLST increased tumor growth in mice. (A-B) Statistical data of mouse tumor weight and volume. (C) Image of mouse tumor tissue (*n* = 6 mice per group). (D) Immunohistochemical was used to detect the expression levels of DLST, HSP70 and SLC31A1 in mouse tumor tissues. Scale bar =100 μm. *P < 0.05, **P < 0.01, ***P < 0.001, ****P < 0.0001 vs. vector group.

In the aforementioned *in vitro* experiments, DLST overexpression increased cuproptosis-related markers and intracellular copper while suppressing apoptosis. To further validate whether this copper-dependent pro-survival effect promotes tumor growth *in vivo*, we established an orthotopic tumor model. To further validate DLST's regulatory role in cuproptosis, we established an orthotopic tumor model in mice. The animal experimental results revealed that tumor weight in the DLST-OE group was significantly larger compared to the vector control group, indicating that overexpression of DLST increased tumor growth in mice (*P*<0.001) (Fig. 4A-C). Furthermore, immunohistochemical analysis of tumor tissues from mice showed that the expression levels of DLST, HSP70, and SLC31A1 were significantly higher in the DLST-OE group than in the vector group, consistent with the aforementioned *in vitro* findings (*P*<0.01) (Fig. 4D).

### EP300 regulates the phosphorylation level of DLST

**Fig. 5:**
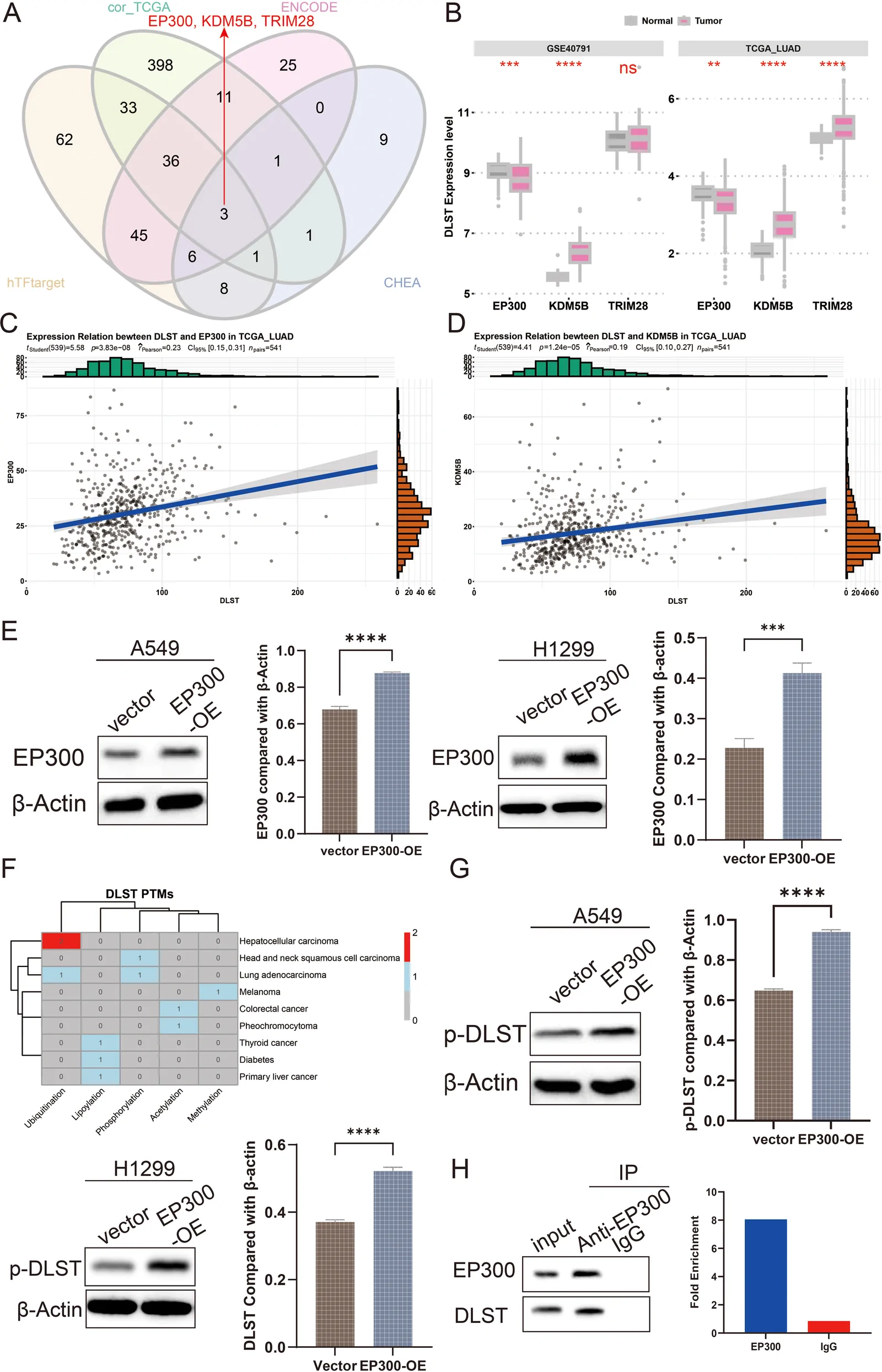
EP300 regulates the phosphorylation level of DLST. (A)Venn diagram of the intersection of hTFtarget, ENCODE, CHEA and TCGA gene expression databases. (B) Differential expression of EP300, KDM5B and TRIM2B in normal tissues and tumor tissues. (C) Correlation between EP300 and DLST expression. (D) Correlation between KDM5B and DLST expression. (E) Effect verification of EP300 overexpression cell lines. (F) Heatmap of DLST post-translational modification. (G) Western blot detection of phosphorylated DLST (p-DLST) in vector and EP300-OE cells. (H) Co-immunoprecipitation (Co-IP) showing EP300–DLST interaction and chromatin immunoprecipitation-qPCR (ChIP-qPCR) showing EP300 enrichment at the DLST promoter in vector and EP300-OE cells, normal rabbit IgG served as a negative control. *P < 0.05, **P < 0.01, ***P < 0.001, ****P < 0.0001 vs. vector group.

To investigate the upstream regulatory molecules of DLST, we conducted a series of systematic bioinformatics analyses and *in vitro* experiments. First, we identified potential transcription factors regulating DLST by analyzing data from the hTFtarget, ENCODE, and CHEA databases, as well as TCGA gene expression correlations. The intersection of these four databases revealed three candidate transcription factors: E1A binding protein P300 (EP300), Lysine demethylase 5B (KDM5B), and tripartite motif containing 2 (TRIM2B) (Fig. 5A). Subsequently, we performed expression analysis of these transcription factors using the GSE40791 and TCGA-LUAD datasets. The results showed no significant difference in TRIM2B expression between normal and tumor tissues in the GSE40791 dataset, while both EP300 and KDM5B exhibited significant expression differences in both datasets (*P*<0.01) (Fig. 5B). We then analyzed the correlation between EP300 and KDM5B with DLST expression, finding that EP300 exhibited a stronger correlation with DLST expression (*P*<0.0001). Therefore, EP300 was selected as the primary focus for upstream regulatory analysis (Fig. 5C-D). To further validate the regulatory role of EP300 in DLST expression, we constructed an EP300-overexpressing (EP300-OE) cell line (*P*<0.001) (Fig. 5E). Additionally, using the PTMD2.0 database, we identified that ubiquitination and phosphorylation modifications of the DLST gene are associated with LUAD (*P*<0.0001) (Fig. 5F). We subsequently performed western blot assays to confirm whether EP300 affects the phosphorylation level of DLST, revealing that p-DLST expression was significantly higher in the EP300-OE group compared to the vector control group (Fig. 5G). Co-IP and CHIP experiments demonstrate that DLST can be directly integrated with EP300 (Fig. 5H). Kaplan-Meier survival analysis further showed that EP300 expression did not reach statistical significance for prognosis (S1A).

### DLST suppressed cell apoptosis by regulating the expression of DLD

**Fig. 6:**
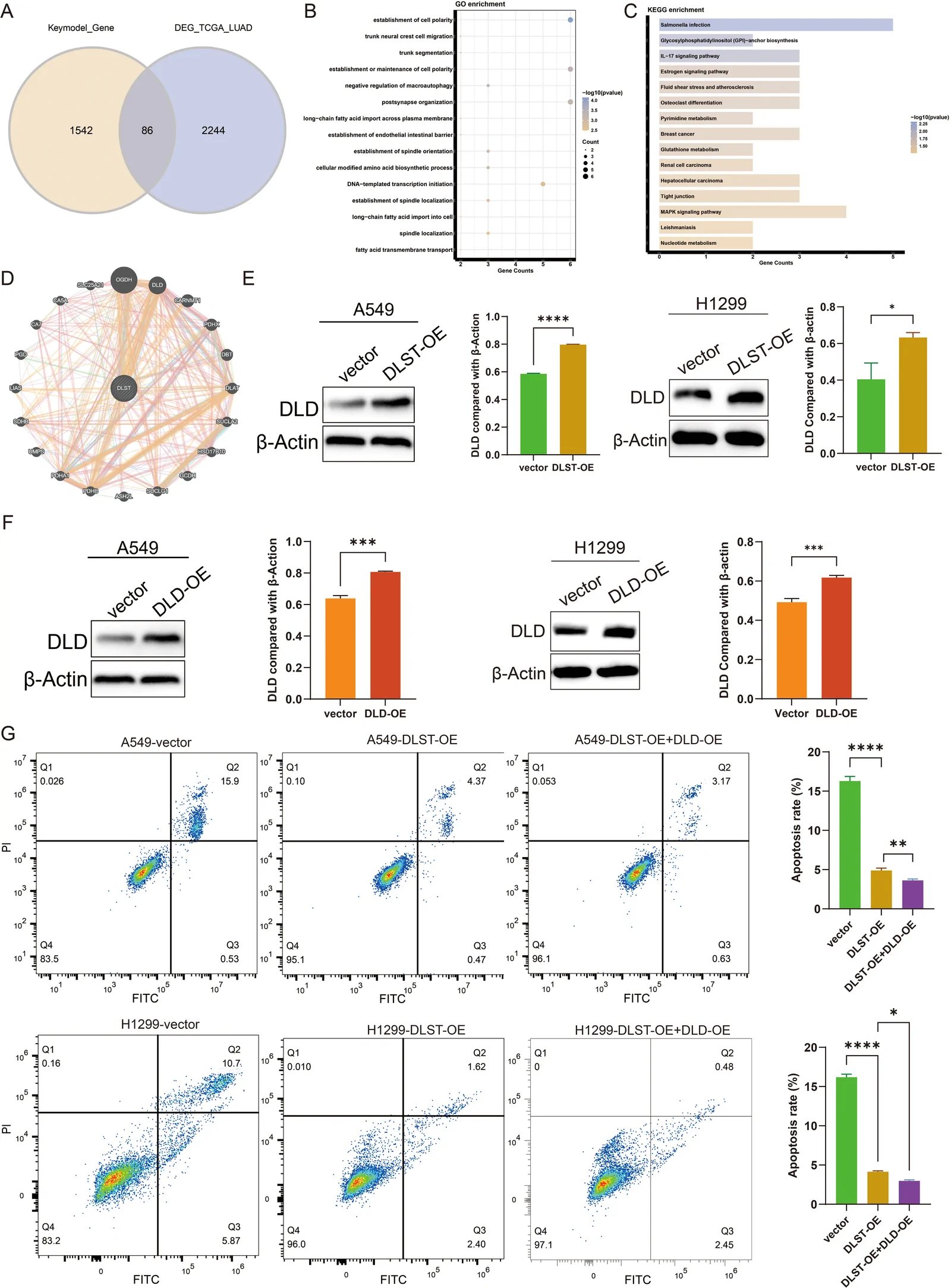
DLST suppressed cell apoptosis by regulating the expression of DLD. (A) Venn diagram of the intersection between DLST-correlated differentially expressed genes and WGCNA key module genes in TCGA-LUAD. (B–C) GO (B) and KEGG (C) enrichment analyses of the intersecting genes. (D) Gene–gene interaction network of DLST with neighboring metabolic genes. (E) Western blot analysis of DLD protein expression in vector and DLST-OE A549 and H1299 cells. (F) Western blot confirmation of DLD overexpression. (G) Flow cytometric quantification of apoptosis in vector, DLST-OE, DLD-OE, and DLST-OE+DLD-OE cells. *P < 0.05, **P < 0.01, ***P < 0.001, ****P < 0.0001 vs. vector group or DLST-OE group.

To investigate the downstream regulatory molecules of DLST, we performed bioinformatic analyses to identify potential downstream regulators. Patients with TCGA_LUAD were divided into high- and low-expression groups, and differentially expressed genes were identified between the two groups. Subsequently, weighted gene co-expression network analysis (WGCNA) was conducted to identify key module genes associated with LUAD (blue module), and the intersection of these genes with the differentially expressed genes yielded 86 critical genes (Fig. 6A). Gene Ontology (GO) analysis revealed that these intersecting genes were primarily associated with "establishment of cell polarity," "postsynaptic organization," and "establishment or maintenance of cell polarity" (Fig. 6B). Kyoto Encyclopedia of Genes and Genomes (KEGG) analysis indicated that the intersecting genes were mainly involved in "Salmonella infection," "Glycosylphosphatidylinositol (GPI)-anchor biosynthesis," and "IL-17 signaling pathway" (Fig. 6C). Gene interaction analysis showed that oxoglutarate dehydrogenase (OGDH), dihydrolipoamide dehydrogenase (DLD), and carnosine N-methyltransferase 1 (CARNMT1) exhibited significant interactions with DLST, with OGDH and DLD displaying particularly close associations (Fig. 6D). Kaplan-Meier survival analysis further showed that high DLD expression was associated with significantly worse overall survival (S1B). To further evaluate the clinical relevance of DLD in LUAD, we examined the expression correlations and prognostic associations among DLST, DLD, and EP300 in the TCGA-LUAD cohort. Pearson correlation analysis revealed significant positive correlations between DLST and DLD, EP300 and DLD, and EP300 and DLST (S1C) Since the interaction between DLST and DLD in LUAD remains unclear, DLD was selected as the primary focus of this study. Western blot analysis demonstrated that overexpression of DLST promoted DLD expression (*P*<0.05) (Fig. 6E). To further explore the regulatory effects of DLST on DLD, we constructed a DLD overexpression (DLD-OE) cell line (*P*<0.001) (Fig. 6F). Flow cytometry results confirmed that overexpression of DLD enhanced the anti-apoptotic effect of DLST on LUAD cells (*P*<0.05) (Fig. 6G). Thus, DLD functions downstream of DLST to amplify the anti-apoptotic signal. This places DLD as a critical link between DLST-mediated metabolic alterations and the suppression of cell death in LUAD.

### DLST and EP300 are mainly distributed in immune related cells

**Fig. 7:**
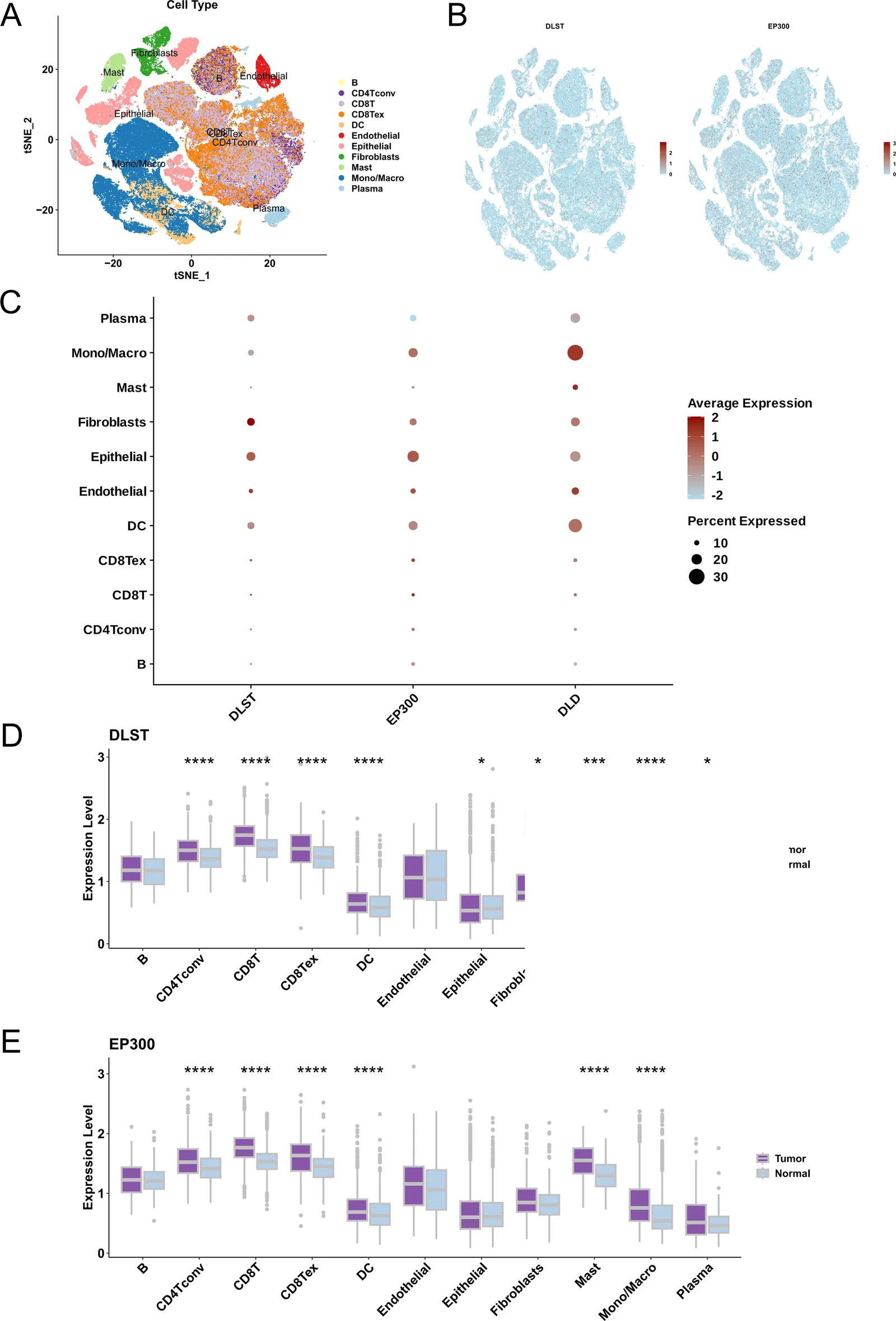
DLST and EP300 are mainly distributed in immune related cells. (A) Cell annotation of GSE131907 dataset. (B-C) Gene cell expression featureplot and dot plot. (D-E) The expression differences of DLST and EP300 between tumor samples and normal samples in each cell.

Although the aforementioned experiments demonstrated the regulatory role of EP300 in DLST, the underlying regulatory mechanism remained unclear. Therefore, we performed single-cell sequencing analysis on the LUAD-related dataset GSE131907. Cell annotation revealed that this dataset primarily consists of B cells, conventional CD4+ T cells (CD4Tconv), CD8+ T cells (CD8T), exhausted CD8+ T cells (CD8Tex), dendritic cells (DC), endothelial cells, epithelial cells, fibroblasts, mast cells, monocytes/macrophages (Mono/Macro), and plasma cells (Fig. 7A). Gene expression featureplot and dot plot revealed that DLST, EP300 and DLD exhibit highly overlapping distribution patterns across these cell types (Fig. 7B-C and S1D). Analyzing the expression differences of DLST, EP300 and DLD between tumor and normal samples across various cell types, we found significant discrepancies in CD4Tconv cells, CD8T cells, CD8Tex cells, mast cells, and Mono/Macro cells (*P*<0.001) (Fig. 7D-E and S1E). These findings imply that EP300, DLST and DLD may regulate each other within immune cells and contribute to LUAD progression through the tumor microenvironment.

### The integrated EP300–DLST–DLD axis score predicts poor prognosis in LUAD

**Fig. 8:**
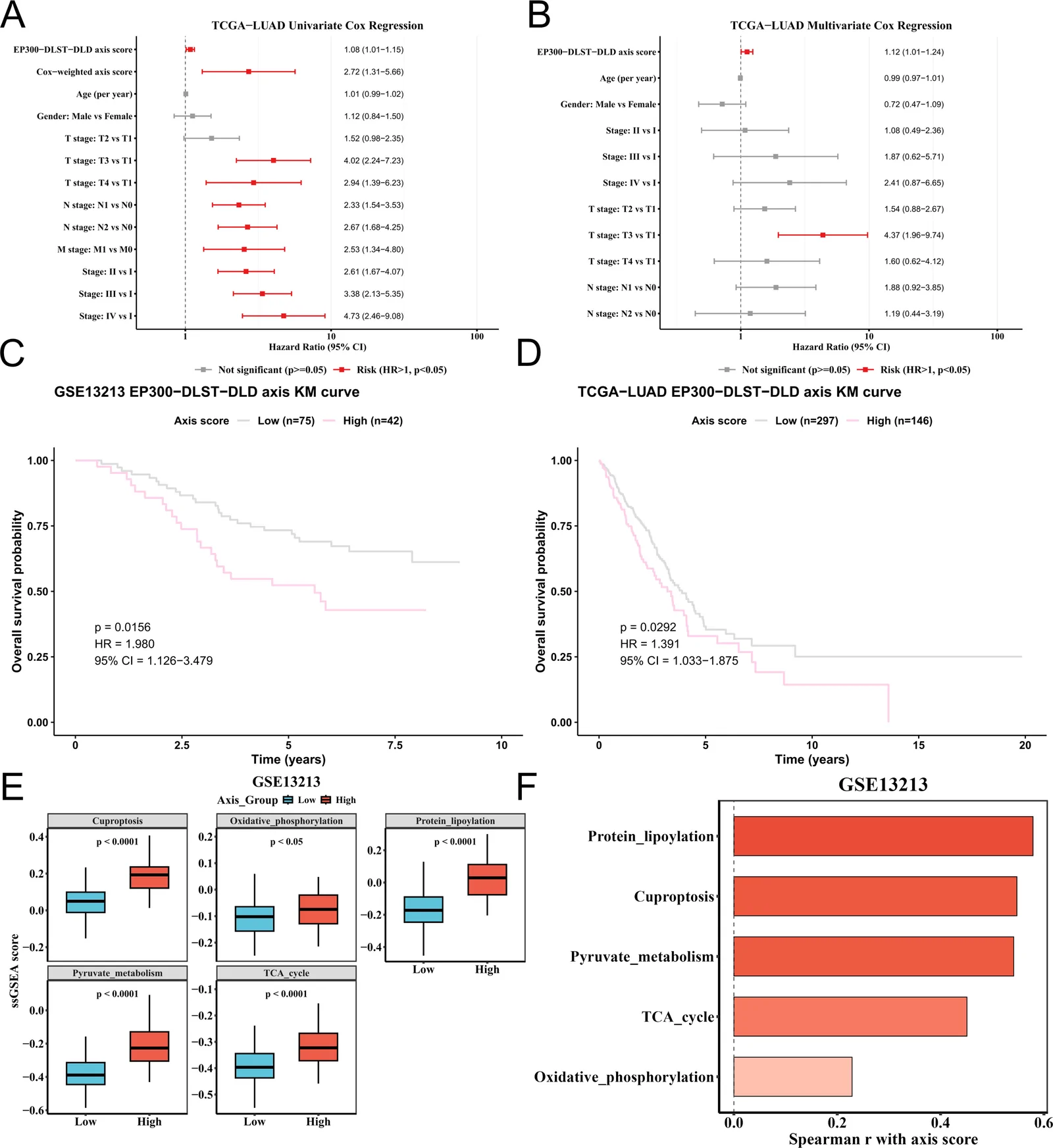
The integrated EP300–DLST–DLD axis score predicts poor prognosis in LUAD. (A) Univariate Cox regression forest plot of the EP300–DLST–DLD axis score and clinicopathological variables in TCGA-LUAD. (B) Multivariate Cox regression forest plot of the axis score in TCGA-LUAD. (C-D) Kaplan-Meier overall survival curves of high- versus low-axis-score groups in the GSE13213 (C) and TCGA-LUAD (D) cohorts. (E) ssGSEA scores of cuproptosis-related and mitochondrial metabolic pathways between high- and low-axis-score groups in GSE13213. (F) Spearman correlation between axis score and pathway scores in GSE13213. *P < 0.05, **P < 0.01, ***P < 0.001, ****P < 0.0001.

Having established that EP300, DLST, and DLD form a functionally connected axis, we next asked whether their combined expression pattern carries prognostic value in LUAD. We constructed an EP300–DLST–DLD axis score by summing the z-scores of the three genes for each patient. Univariate Cox regression analysis in TCGA-LUAD showed that the axis score was significantly associated with overall survival, and a Cox-weighted score showed an even stronger association (Fig. 8A). Importantly, multivariate Cox regression analysis adjusted for age, gender, and tumor stage demonstrated that the axis score remained an independent prognostic factor (Fig. 8B). Kaplan-Meier analysis further confirmed that patients with a high axis score had significantly worse overall survival than those with a low score in both TCGA-LUADand the independent GSE13213 validation cohort (Fig. 8C-D). To explore the biological processes associated with this axis, we performed single-sample gene set enrichment analysis (ssGSEA) in the GSE13213 cohort. Patients with a high axis score showed significantly higher activity of cuproptosis-related, protein lipoylation, pyruvate metabolism, TCA cycle, and oxidative phosphorylation pathways (Fig. 8E). Consistently, axis score displayed positive Spearman correlations with all five pathways, with the strongest associations observed for protein lipoylation and cuproptosis (Fig. 8F). These results suggest that the integrated EP300–DLST–DLD axis not only predicts clinical outcome but also reflects a metabolic state characterized by cuproptosis-associated and mitochondrial TCA-cycle signaling.

## Discussion

LUAD, the most prevalent subtype of non-small cell lung cancer (NSCLC), poses a significant threat to human survival. Gaining an in-depth understanding of its pathogenesis is crucial for addressing the challenges in achieving a cure. Cuproptosis, a type of programmed cell death that has garnered increasing attention in recent years, has been extensively studied in the context of LUAD. The present study investigates the biological functions of the DLST gene, particularly its role in regulating cuproptosis and its potential influence on the progression of lung adenocarcinoma. Furthermore, through a combination of bioinformatics analysis and *in vitro* experiments, we have identified and validated the regulatory role of EP300 in controlling DLST expression.

In this study, we found that DLST overexpression simultaneously elevates cuproptosis-related markers (HSP70, SLC31A1) and intracellular copper while promoting tumor cell survival and growth. Cuproptosis is defined as a mitochondria-dependent form of programmed cell death driven by copper-mediated aggregation of lipoylated TCA-cycle proteins. Therefore, increased marker expression alone should not be equated with activated cuproptotic cell death. In our LUAD model, reduced apoptosis, enhanced DLD expression, and accelerated tumor growth indicate that DLST channels copper stress toward a pro-survival program rather than executing cell death. HSP70 is a stress-inducible chaperone that can protect cells against proteotoxic damage, and SLC31A1-mediated copper import may serve as a metabolic signal that is counterbalanced by DLST-driven DLD upregulation. Thus, we propose that DLST remodels copper homeostasis and cuproptosis-associated signaling to increase cellular copper tolerance, thereby supporting tumor progression. Future studies using cuproptosis-specific inducers and direct cell-death assays are required to formally test whether DLST sensitizes or protects LUAD cells against cuproptosis.

Several cuproptosis-related genes have been implicated in the progression of lung adenocarcinoma. Previous studies have largely focused on constructing prognostic signatures based on cuproptosis-related gene panels. For example, Ma et al. developed a prognostic model incorporating multiple cuproptosis-related genes and identified DLAT and DLST as predictors of metastasis in non-small cell lung cancer [22]. Similarly, Liang et al. established a deep learning-based model using cuproptosis-related genes for prognostic evaluation of LUAD [23]. These studies demonstrate the clinical relevance of cuproptosis-related genes but provide limited mechanistic insight into how individual genes regulate LUAD progression. Li et al. reported that DLD is associated with poor prognosis and malignant biological characteristics in LUAD [24], yet the upstream regulatory mechanisms and functional partners of DLD in this context remain poorly characterized. In contrast to these correlation-based studies, this work elucidates a regulatory axis linking EP300-mediated DLST phosphorylation to DLD upregulation and apoptosis suppression in LUAD. We demonstrate that DLST not only promotes the expression of cuproptosis-related proteins, including HSP70 and SLC31A1, but also increases intracellular copper levels and inhibits apoptosis, effects that are partially reversed by the copper chelator TTM. Moreover, our identification of EP300 as an upstream regulator of DLST phosphorylation adds a previously unrecognized layer of post-translational regulation to copper metabolism in LUAD.

To interpret our findings in simple terms, we propose a sequential model. First, EP300 interacts with DLST and enhances its phosphorylation, which may increase DLST stability or activity. Second, activated DLST alters intracellular copper handling, leading to copper accumulation and increased expression of HSP70 and SLC31A1. These changes likely represent a copper stress response rather than execution of cuproptosis. Third, DLST upregulates DLD, which in turn suppresses apoptosis. The final outcome is not copper-induced cell death but enhanced survival of LUAD cells, which translates into accelerated tumor growth *in vivo*.

EP300, a histone acetyltransferase (HAT), is encoded by the EP300 gene in humans. As a transcriptional coactivator, it plays a crucial role in regulating various biological processes. Leveraging its inherent acetyltransferase activity, EP300 influences cancer progression by modifying downstream molecules through post-translational acetylation. For instance, EP300 promotes ferroptosis in pancreatic cancer cells by acetylating HSPA5 at the K353 site [25]. Additionally, it inhibits the JAK/STAT pathway by acetylating SOCS1 at H3K27ac, providing protective effects against coronary artery disease [26]. Furthermore, EP300 interacts with REST to upregulate KIF15 expression, facilitating the progression of glioblastoma cells from the S phase to the G2 phase and promoting tumor development [27]. Given its critical role in cell cycle regulation, research on EP300 as a therapeutic target has commenced. CCS1477, an EP300 inhibitor, suppresses MYB acetylation at H3K27 in myeloid leukemia, thereby inhibiting disease progression [28]. Another EP300 inhibitor, SGC—CBP30, has shown promising clinical effects in acute liver failure by modulating the P38-JNK pathway, reducing inflammation and oxidative stress in intestinal epithelial cells, and protecting against epithelial damage [29]. Interestingly, while EP300 promotes disease progression in hematologic malignancies, acute liver failure, and glioblastoma, it exhibits tumor-suppressive functions in certain cancers. Overexpression of EP300 in ovarian cancer cells induces cell cycle arrest at the G1 phase, thereby impeding tumor growth [30].

Our findings demonstrated that EP300 overexpression enhances DLST phosphorylation, which may contribute to the suppression of apoptosis and promotion of LUAD progression. However, EP300 mutations are frequently observed in various cancers. For example, high-frequency nucleotide mutations of EP300 have been reported in gastric and colorectal cancers [31], while in esophageal squamous cell carcinoma, frameshift or truncating mutations of EP300 occur in 17% of cases [32]. These findings suggest that EP300 mutations may disrupt its tumor-suppressive functions. In summary, EP300 exhibits dual roles in cancer, likely due to its acetyltransferase activity, transcriptional regulatory capabilities, and high mutation frequency across cancers. These characteristics underscore its strong context-specificity and complexity in different tumor types. Understanding EP300′s distinct roles in various cancers is essential for unraveling its mechanisms and advancing personalized therapies for cancer patients.

To gain a deeper understanding of the mechanism by which DLST inhibits apoptosis, we screened and verified downstream regulatory molecules of DLST, discovering that DLD is a downstream effector of DLST. Recent studies have implicated a pivotal role of DLD in cuproptosis. During cuproptosis, DLD primarily binds copper ions, leading to abnormal protein aggregation and loss of function, thereby facilitating the progression of the copper death process [33]. Numerous studies have demonstrated DLD's involvement in disease development and advancement across multiple pathological conditions. For instance, DLD is highly activated in spinal cord injury tissues, where its mechanisms primarily involve interactions between cuproptosis and the immune microenvironment [34]. Pan-cancer analyses have revealed that DLD exhibits strong tissue-specific expression patterns across cancers. In breast cancer, colon adenocarcinoma, and renal clear cell carcinoma, DLD expression is significantly higher in normal tissues compared to tumor tissues, and its high expression correlates with better prognosis. Conversely, in ovarian cancer and endometrial cancer, DLD expression is significantly higher in tumor tissues than in normal tissues, and its high expression is associated with poorer outcomes [[35], [36], [37]]. In pulmonary diseases, DLD has been implicated in disease progression. Clinical and animal experimental data on chronic obstructive pulmonary disease (COPD) demonstrate that DLD is abnormally overexpressed in both COPD patients and animal models, suggesting its potential role in disease development [38]. Public database analyses indicate that high DLD expression in lung adenocarcinoma tissues is positively correlated with poorer patient prognosis. *In vitro* experiments have shown that DLD deficiency impairs the migratory phenotype of lung adenocarcinoma cells [24].

Given DLD's significant role in lung adenocarcinoma, researchers have developed a deep learning predictive model incorporating DLD as a key factor. This model demonstrates high predictive accuracy, offering a novel approach for the early diagnosis of LUAD [23]. Previous studies have shown a positive correlation between DLST and DLD expression in LUAD, consistent with our findings [37]. Additionally, high expression of DLST and DLD has been identified as a significant predictor of metastasis in NSCLC [22]. Similarly, our research demonstrates that overexpression of DLST inhibits apoptosis in lung adenocarcinoma cells, and this effect is further enhanced by DLD overexpression.

Despite the insights provided by this study into the biological functions of DLST and its upstream and downstream regulatory molecules in lung adenocarcinoma, there are areas that warrant further refinement in terms of resource utilization, methodology, and research depth. First, and most importantly, the current study lacks direct immunohistochemical or protein-level validation in patient-derived LUAD tissue specimens. While our bioinformatic analyses using TCGA and GEO datasets revealed elevated DLST mRNA expression in LUAD tissues and its association with poor prognosis, and while our functional experiments in LUAD cell lines and orthotopic mouse models demonstrated that DLST promotes tumor growth by regulating cuproptosis and apoptosis, these lines of evidence are preclinical. We did not perform immunohistochemical staining or Western blot analysis of DLST, DLD, EP300, HSP70, or SLC31A1 in primary human LUAD tissues, nor did we correlate protein expression levels with patient survival, TNM stage, lymph node metastasis, or tumor differentiation. Consequently, the translational relevance of the EP300-DLST-DLD axis in clinical LUAD remains to be established. Future work should validate these findings in a large, independent LUAD patient cohort with paired adjacent normal tissues, using standardized IHC scoring and multivariable survival analysis. Second, the functional evidence is based primarily on DLST overexpression in two LUAD cell lines and an orthotopic mouse model. Although these gain-of-function experiments consistently indicate a pro-survival and pro-tumorigenic role for DLST, they do not formally establish that endogenous DLST is required for LUAD progression. Loss-of-function approaches, including siRNA/shRNA-mediated knockdown or CRISPR/Cas9-mediated knockout of DLST, are therefore needed to confirm causality and to exclude potential off-target or artifactual effects associated with overexpression. Third, the depth of investigation into DLST-regulated downstream pathways is insufficient. While this study identified DLD as a downstream effector and validated its role in inhibiting apoptosis in synergy with DLST, we did not further explore the signaling pathways modulated by DLST, such as ROS accumulation, mitochondrial membrane potential, and copper homeostasis. Additionally, the absence of large-scale transcriptomic profiling (RNA-seq) or phosphoproteomics data hinders a systemic understanding of the DLST-regulated signaling network. In summary, future work should focus on expanding clinical sample validation, confirming the direct interaction between EP300 and DLST, and conducting a comprehensive investigation of the signaling pathways mediated by DLST to better elucidate its role in cuproptosis regulation in LUAD.

The integrated EP300–DLST–DLD axis score developed in this study may offer practical value for LUAD risk stratification. In TCGA-LUAD, the axis score remained an independent prognostic factor after adjustment for age, sex, and tumor stage, and its prognostic value was successfully validated in the independent GSE13213 cohort. These findings suggest that a three-gene expression signature could be incorporated into existing prognostic models to identify high-risk LUAD patients who might benefit from more intensive postoperative surveillance or adjuvant therapy after surgical resection. From a biomarker perspective, individual components of the axis, particularly DLST and DLD, could be evaluated as protein markers in formalin-fixed tumor tissue or, with further analytical validation, in liquid biopsies. However, given the context-dependent roles of EP300 and the narrow therapeutic window of copper metabolism modulation, any clinical application would require rigorous preclinical optimization and patient stratification based on axis score. The most immediate next validation step is to confirm the axis score and protein expression patterns in an independent, clinically annotated LUAD cohort with paired adjacent normal tissue and complete follow-up data, using standardized immunohistochemical scoring and multivariable survival analysis.

## Conclusion

**Fig. 9:**
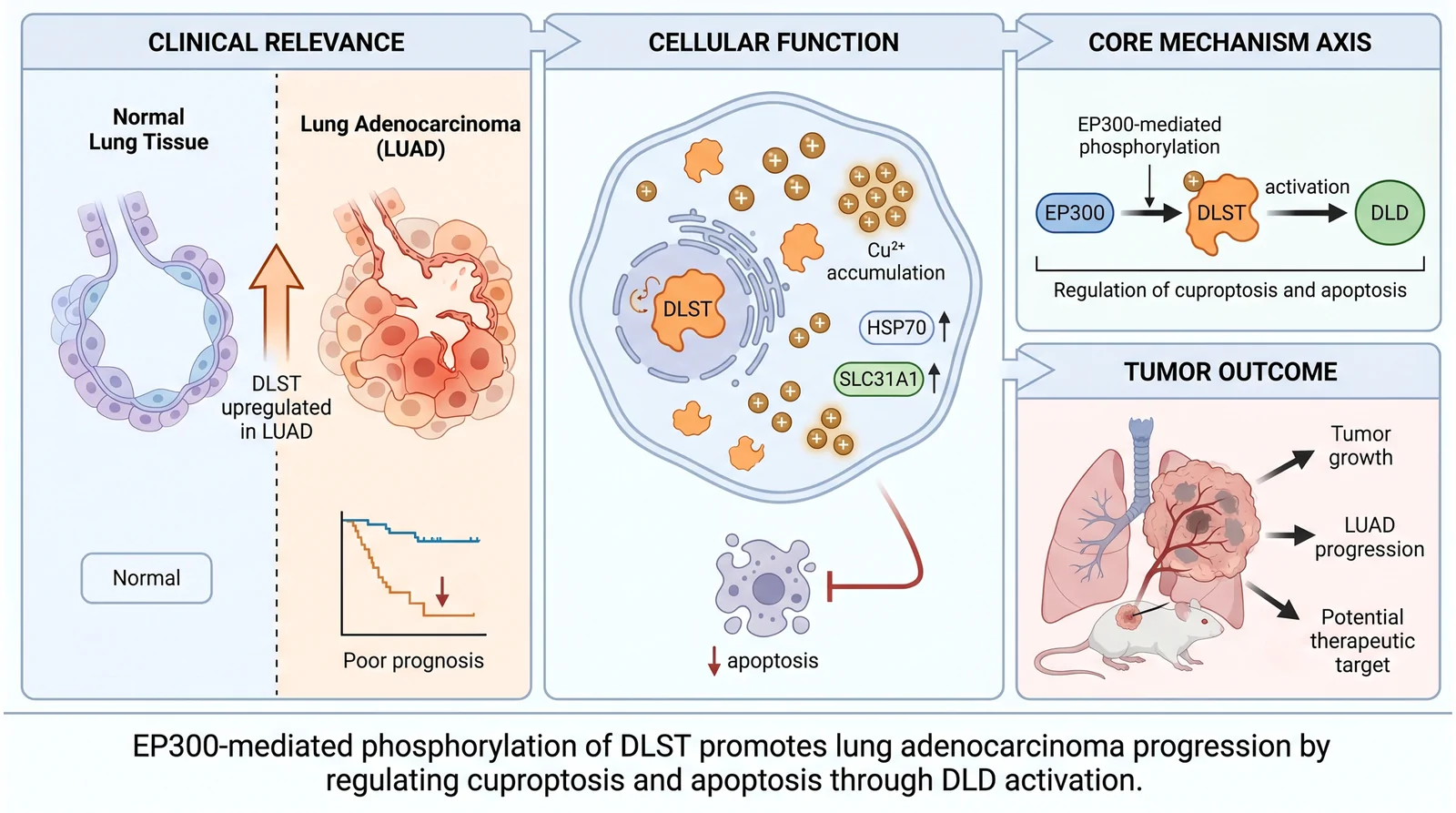
Schematic diagram of the mechanism of action of the EP300-DLST-DLD axis in lung adenocarcinoma. EP300 enhances DLST phosphorylation, driving intracellular copper accumulation via upregulated SLC31A1/HSP70, which suppresses apoptosis, promotes tumor growth, and correlates with poor prognosis.

DLST is upregulated in lung adenocarcinoma and correlates with poor prognosis. It promotes tumor progression by regulating copper homeostasis, increasing cuproptosis-related protein expression, and inhibiting apoptosis. Mechanistically, EP300 enhances DLST phosphorylation, while DLD mediates its downstream anti-apoptotic effects. *In vivo* data confirm that overexpression of DLST increased tumor growth in mice. Additionally, DLST and EP300 are co-expressed in immune-related cells, suggesting a role in the tumor microenvironment and identifying a potential therapeutic axis in LUAD (Fig. 9).

## Ethics approval and consent to participate

All procedures were ratified by the Ethics Committee of Shenzhen People’s Hospital (2023-245-01) and conducted as per the *Declaration of Helsinki.* Signed informed consent was received from each eligible participant.

## Consent for publication

Not applicable.

## Clinical trial number

NA.

## Funding

This study was supported by Natural Science Foundation of Shenzhen (NO. JCYJ20230807112401002).

## CRediT authorship contribution statement

**Tonghai Huang:** Writing – review & editing, Writing – original draft, Visualization, Validation, Software, Resources, Project administration, Investigation, Funding acquisition, Data curation, Conceptualization. **Lin Chen:** Writing – review & editing, Validation, Supervision, Resources, Methodology, Formal analysis, Conceptualization. **Zeyao Li:** Supervision, Software, Project administration, Formal analysis, Conceptualization. **Xiean Ling:** Writing – review & editing, Supervision, Resources, Methodology, Conceptualization. **Kangqi Ren:** Supervision, Project administration, Formal analysis, Data curation.

## Declaration of competing interest

All authors declare that there is no conflict of interests in this study.

## Data Availability

All the data generated or analyzed during this study are included in this published article. Data can be requested from the author upon reasonable request.

## Appendix Supplementary materials

Supplementary 1. (**A**) Kaplan-Meier overall survival curves for EP300 and (**B**) DLD. (**C**) Pearson correlation analyses of DLST, EP300, and DLD expression in TCGA-LUAD. (**D**) UMAP plot showing DLD expression in the GSE131907 single-cell dataset. (**E**) Box plots comparing DLD expression levels between tumor and normal samples within each cell type. **P* < 0.05, ***P* < 0.01, ****P* < 0.001, *****P* < 0.0001. Image, application 1

Image, application 2
