# Supplementary material for: DLST drives lung adenocarcinoma progression by modulating cuproptosis-related copper homeostasis and apoptosis via an EP300-dependent mechanism
Source: Transl Oncol. 2026 Aug 14;72:102975. doi: 10.1016/j.tranon.2026.102975 (PMC13503093; doi:10.1016/j.tranon.2026.102975)
Supplement: Supplementary file 1 [file mmc1.docx]

Figure 6E

Figure 6G

Figure 5F

Figure 5E

Figure 2A

Figure 2C

Figure 6H

Figure 3A
